# A scoping review of mobile apps for sleep management: User needs and design considerations

**DOI:** 10.3389/fpsyt.2022.1037927

**Published:** 2022-10-18

**Authors:** Abdullah Al Mahmud, Jiahuan Wu, Omar Mubin

**Affiliations:** ^1^Centre for Design Innovation, Swinburne University of Technology, Hawthorn, VIC, Australia; ^2^School of Computer, Data and Mathematical Sciences, Western Sydney University, Penrith, NSW, Australia

**Keywords:** mobile apps, human computer interaction, sleep disorder, sleep management, sleep apnea

## Abstract

Sleep disorders are prevalent nowadays, leading to anxiety, depression, high blood pressure, and other health problems. Due to the proliferation of mobile devices and the development of communication technologies, mobile apps have become a popular way to deliver sleep disorder therapy or manage sleep. This scoping review aims to conduct a systematic investigation of mobile apps and technologies supporting sleep, including the essential functions of sleep apps, how they are used to improve sleep and the facilitators of and barriers to using apps among patients and other stakeholders. We searched articles (2010 to 2022) from Scopus, Web of Science, Science Direct, PubMed, and IEEE Xplore using the keyword sleep apps. In total, 1,650 peer-reviewed articles were screened, and 51 were selected for inclusion. The most frequently provided functions by the apps are sleep monitoring, measuring sleep, providing alarms, and recording sleep using a sleep diary. Several wearable devices have been used with mobile apps to record sleep duration and sleep problems. Facilitators and barriers to using apps were identified, along with the evidence-based design guidelines. Existing studies have proved the initial validation and efficiency of delivering sleep treatment by mobile apps; however, more research is needed to improve the performance of sleep apps and devise a way to utilize them as a therapy tool.

## Introduction

Sleep is an essential biological need for human beings that will support us in getting resting, healing, and being ready for the next day. It is widely accepted that disturbed sleep is an influential factor leading to many mental health disorders. According to the Sleep Health Survey of Australian Adults (1), inadequate sleep has affected 33–45% of adults in Australia. Sleep disorders include short sleep duration, insomnia, snoring, sleep apnea, parasomnias, and restless leg syndrome. The treatment of sleep disorders varies, and some can be delivered online, such as Cognitive Behavioral Therapy for insomnia. With the development of technology, there are fewer barriers to accessing mobile phones, and we can establish mobile apps with many useful functions. Thus, mobile apps have become a popular tool for delivering sleep treatments. For example, with the microphone and sound sensor of the mobile phone, we can monitor people's breath while sleeping. It is crucial to analyse the utilization of mobile apps to support sleep and further improve the quality of daily life.

Previous studies have already analyzed the validation and efficiency of sleep apps. For example, Baron et al. (2) conducted a scoping review of the use of consumer-targeted wearable and mobile technology. They found that most of the articles they reviewed focused on validation of sleep application, and there was a gap in interventions in more target populations such as patient populations (2). Shin et al. (3) stated that mobile phone interventions could attenuate sleep disorders and improve sleep quality. With a three-piece test set up by Stippig et al. (4), the result shows that most apps cannot distinguish and record snoring noises from various disturbing noises in real-life situations. Cajita et al. (5) also stated in their scoping review that the utility of wearable activity monitors in improving sleep needs more evidence to support it. An app review completed by Choi et al. (6) found that most sleep apps in the market cannot meet the quality, content, and functionality requirements to manage sleep by users. Therefore, guidelines are needed to improve the performance of sleep apps, and this paper aims to review the current apps to understand the research gaps and provide guidelines to design better sleep apps. We have conducted a scoping review of the articles published in 2010–2022 to understand the features of the sleep apps, user requirements and the design guidelines to improve those apps.

## Methodology

We applied the scoping review methodology proposed by Arksey and O'Malley (7), which had been further improved by the methodology developed by the Joanna Briggs Institute (8).

### Stage 1: Identifying the research questions

In this step, we reviewed previous research works and determined the gaps. The research questions are as follows:

1. What are the key functions of the current sleep apps?
2. What are the limitations of sleep apps?
3. What are the user requirements and design guidelines of sleep apps?

### Stage 2: Identifying relevant studies

We used keywords such as sleep apps and sleep monitoring. To find relevant articles, we searched Scopus, Web of Science, Science Direct, PubMed, and IEEE Xplore. Only peer-reviewed articles written in English and published from 2010 to 2022 were considered.

### Stage 3: Study selection: Inclusion and exclusion criteria

After exporting research results into Endnote and deleting the duplication, we screened the titles and abstracts of all these articles and removed those unrelated to our topic. The following inclusion criteria were followed: (1) Does the article involve a sleep app? (2) Does the article report the testing of a sleep app? and (3) Is the article written in English?

**Figure 1:**
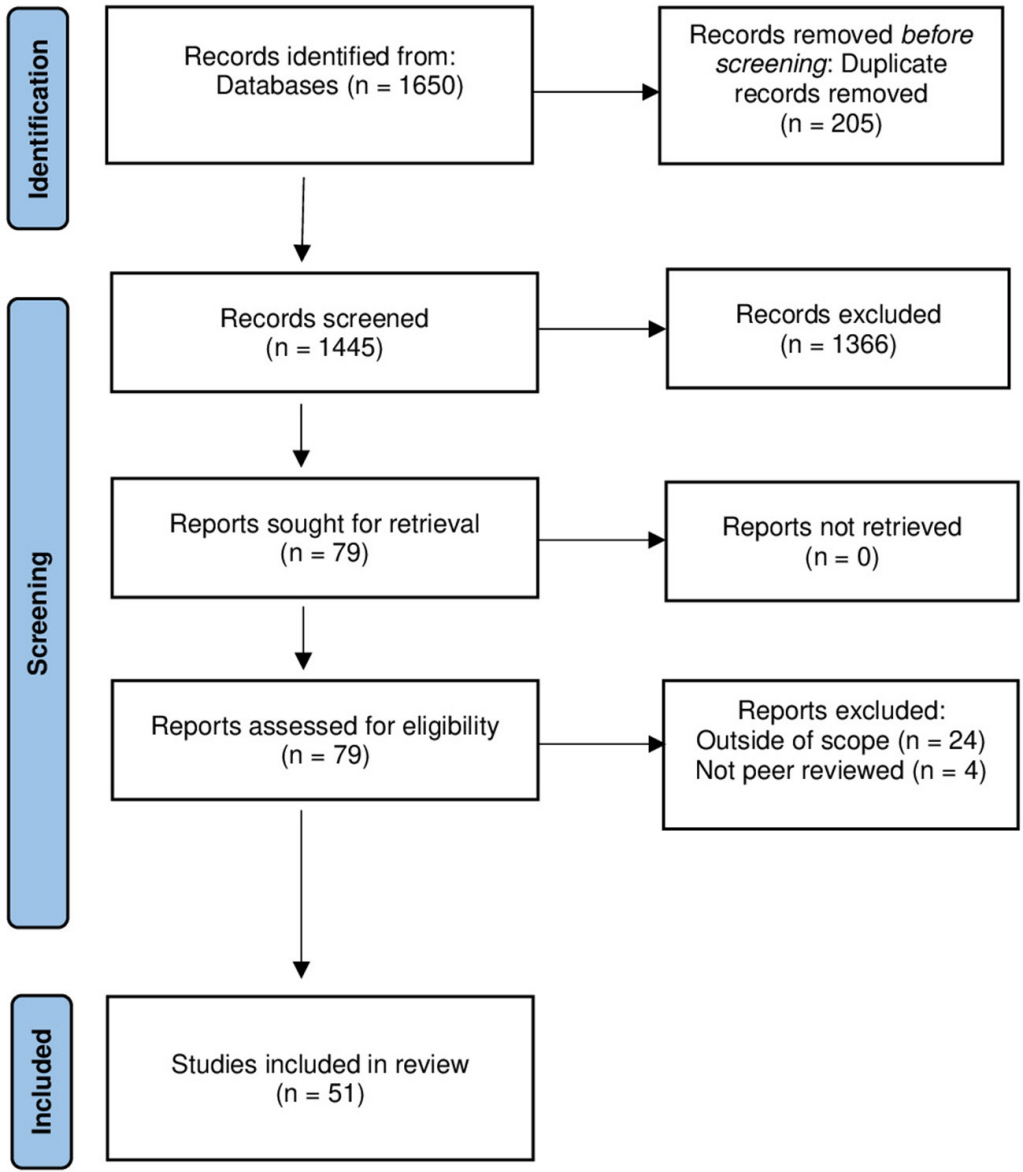
PRISMA diagram showing the article selection process.

A full-text review was carried out for these references, and those only 51 articles were selected to extract data for analysis. More detailed information can be found in Figure 1.

### Step 4: Charting the data

All included studies were reviewed and charted using a data extraction sheet. The details about the articles such as the country of publication, research method, year of publication, target group, target sleep issue, and the information about the functions provided by the sleep apps, the limitation and outstanding of sleep apps, the user requirement of the app and the design guidelines were extracted for analysis.

### Stage 5: Collating, summarizing, and reporting the results

The filtered studies were analyzed, and the details were presented using tables and graphs.

## Results and discussion

### Overview of the studies

In total, 51 articles were included in the final review. Thirty-three percentage (17/51) of these publications come from the United States, 20% (10/51) from the UK, 12% (6/51) from China, 10% (5/51) from Canada, 8% (4/51) from Korea, 6% (3/51) from Ireland and 6% (3/51) from Austria. Thirty-one percentage (16/51) of the articles did not focus on one particular issue of sleep disorders or changing sleep habits. Only 20% (10/51) of them are related to apnoea issues, 12% (6/51) of them aimed to help people to develop healthy sleep habits, 22% (11/51) tried to treat insomnia and 12% (6/51) focused on recording sleep and wake detection. Two articles focused on sleep onset and utilized external stimuli to help people fall asleep faster.

### Functions of the current sleep apps

Sixty-six percentage (34/51) articles have mentioned the monitoring functions of mobile sleep applications, indicating that monitoring sleep is the most common function among the existing applications (9–11). Rönkkö (12) has connected mobile apps with an activity tracker; thus, the data collected by the external tracker can be transferred to the mobile app. The mobile app has several functions such as monitoring, reminding, alarm and goal setting. Validated smartphone apps can help detect sleep times and related sleep issues to support clinical treatment. Such a treatment can be provided by a smartphone app called UP! that is capable of accurately measuring sleep durations for individuals with bipolar disorder (13). Non-invasive sleep monitoring using Ballistocardiography (BCG) can help people wake up smoothly compared to Polysomnography (PSG) (14). In addition, a smartphone meditation app can help the prehypertensive population to measure sleep quality (15).

According to the data extracted from these 51 articles, an alarm is the second most frequent function. The concept of alarm in different apps can sometimes have different meanings. Firstly, it is the regular clock that users need to set a time in the app in advance, and then the app will start an alarm at that time. In this case, users determine the time when to awake. Some apps monitor users' sleep and measure light and deep sleep so that the app will awaken users in light sleep (16). In this case, the app will determine the time to awake users.

CBT-I Coach is a mobile application that delivers cognitive behavioral therapy for insomnia (17–19). These apps offer sleep diaries, education, alarm and relaxation exercise functions. The app enables insomnia patients to update their sleep prescription from CBT-I providers to the app, and then the app will help manage and remind users of their own needs, such as recommended bedtime and wake time. Users can also export their sleep diary for further usages, such as translation, to health professionals for treatment.

Several other apps have been developed to support people with insomnia. For instance, researchers developed a mobile app to deliver Sleep restriction therapy (SRT) for people with insomnia (20). Another mobile app called “MIND MORE” can help with the self-management of insomnia (21). Similar to “MIND MORE,” “Insomnia Coach” app helps people to self-manage insomnia (22). KANOPEE app provides behavioral intervention for individuals with insomnia symptoms through an interaction with a virtual agent (23). This app provides more benefit than an electronic sleep diary to support people with insomnia.

Sleep quality and quantity are two factors for measuring sleep. Most mobile apps are implemented by consumer sleep tracking devices to collect and utilize data to measure users' sleep quality and quantity (24). The measurement accuracy of these tracking devices and mobile apps is always low compared to traditional polysomnography, the gold standard for sleep assessment. The current technology cannot support mobile apps as accurate as polysomnography; however, it can be a suitable replacement because of its high cost and low convenience (25). Researchers have developed an EarlySense contact-free sensor and smartphone app to collect vital signs and analyse sleep patterns (26). Validation of the EarlySense sensor showed accuracy in detecting sleep and wake states relative to the gold standard polysomnography.

Sleep disorder detection is one crucial function provided by mobile apps (27). Disorders in the early stage are much easier to be treated than later. Tseng et al. (28) and Behar et al. (29) developed an intelligent mobile app to screen users and detect obstructive sleep apnoea patients. The SleepAp (29) uses signal processing and machine learning algorithm to screen for obstructive sleep apnoea at a negligible cost. Researchers also developed a smartphone app called “Firefly” to measure obstructive sleep apnoea (30). The app is reliable and accurate in detecting obstructive sleep apnoea compared to polysomnography.

With the internet, mobile apps can enable users in the same community and share information and data. Being in the community can be achieved within users and also external identities. Users can share their sleep goals and get competed in the app presented by Rönkkö (12), and they can also export the data from mobile apps and transfer it by a network to other individuals such as health care providers (18).

### Limitations of sleep apps

The most significant limitation of sleep apps is the accuracy to monitor sleep (*n* = 5). Mansukhani and Kolla (31) evaluated the major shortcoming and limited utility of sleep apps in the clinical population. They concluded that sleep data gathered from tracking devices are less reliable in patients with insomnia and fragmented sleep problems. The tracking devices accommodated with sleep apps could not distinguish various sleep stages in different users (31). Several smartphone applications were developed for sleep–wake detection through sound and movement sensors (32). While comparing the performance of the apps, the sleep wake detection was found not sufficiently reliable compared with polysomnography. Wearable devices have been used with a mobile app to record sleep; however, they have shortcomings in measuring sleep problems (33). For instance, ActiGraph wGT3X-BT accelerometers have been used with the SleepBot app for people with schizophrenia to measure sedentary behavior and sleep. It was observed that the app's measure of sleep was inaccurate. Smartphone applications and associated wearable sleep tracking devices have limitations in detecting sleep durations, efficiency, and sleep cycle detection sleep compared to polysomnography (PSG) (34). Therefore, to improve the accuracy of sleep predictions, researchers suggest combining actigraphy-based sleep detection by using the data from movement sensors with the use of technology detected by smartphones (35).

The issue of poor sleep cycle detection by smart phone app has been reported in Bhat et al. (36) and the results suggest that current sleep apps need to have improved accuracy to be used as potential clinical utility tool.

While mobile apps collect and manage users' sleep data, the privacy of the data becomes a common concern. Leigh et al. (37) assessed the quality of apps designed for chronic insomnia disorders from the Android Google Play Store and evaluated their risk along with the privacy policy. Fino et al. (32) compared the performance of four existing sleep apps with polysomnography. The result showed that none of these four apps could detect rapid eye movement sleep, and the overall performance of sleep apps was worse than polysomnography. Short battery life is also a limitation of sleep apps, especially for those accommodated with small sensor devices (38).

### User requirements and design approaches to improve the performance of sleep apps

Different user populations will have different characteristics regarding sleep, and each individual will further require unique demands from sleep apps. User-centered design is an efficient approach to developing clinical applications proved by Luna et al. (39) and McCurdie et al. (40). Aji et al. (41) applied a mixed-method study to explore and determine the end users' requirements and preferences for sleep applications. Users prefer the free app so that they can make a long-term commitment. Personalisation is an essential requirement for users to sleep apps. Nguyen (42) presented an approach to personalizing smart apps by personality traits and chorotype. Users are more likely to choose an app that provides a privacy policy with a high-security level (28).

Mobile apps can be used to support healthy sleep habits. Grigsby-Toussaint et al. (43) examined 35 apps and found that only a few apps included features to change behavior.

Theory-based, evidence-based and user-based are the three approaches to develop sleep apps as observed in those articles. Antezana et al. (44) evaluated thirty existing apps for physical activity, diet and sleep and determined whether they had followed theory-based behavior change techniques. All the 30 apps included at least one behavior change technique (BCT) in their design, and the most frequently used ones were goal setting and feedback.

User-centered is one of the basic design method for developing sleep apps (41). The preferences and social and cultural contextual factors of target populations should be considered when designing sleep apps (45). Evidence-based principles are also frequently included in app designs (46). Users preferred the apps that provide sleep tips based on empirical evidence (47). Personalized feedback (48), connection to other apps and multifunctional (49), and engaging content and easy-to-follow format (46) are some other design guidelines included in the articles. To engage the users, the app's content can be more interactive, such as a virtual pet (50).

The apps that automatically track sleep should enable users to edit the records manually, let the users take control of their data, and enable them to export the data (6). Hosszu et al. (51) stated that sleep apps should connect users with medical professionals and consider ethical issues when designing. Shin et al. (3) and Fino and Mazzetti (52) also suggested developing apps following design guidelines that are evidence-based and include behavior change techniques. Table 1 summarizes the key features of sleep apps and design considerations found in our included studies.

**Table 1:** Some key features of sleep apps and design considerations.

**Sleep app features**	**Frequency**	**App design considerations**	**Frequency**
Sharing information: being part of a social community	4	Personalisation	4
Instant feedback	4	Simple device	2
Education	3	Evidence-based	10
Monitor	34	Theory-based	6
Alarm	5	Low cost	1
Relaxation exercises	3	Accessible format	1
Measure sleep	6	Easy to use	2
Diary	5	Engagement	5
Sleep disorder screen	5	User control	1
Recommend ideal sleep time	2	User centered	3
Sleep–wake detection	2	Social connectedness	4

## Conclusion

Mobile apps have become a popular way to support sleep, and many such apps exist in the market. The most frequently provided functions by the apps are sleep monitoring, measuring sleep, providing alarms, and recording sleep using a sleep diary. There is a lack of apps that support active medical therapy. Therefore, the role of sleep apps in supporting sleep disorder treatments still needs further investigation. Easy-to-use, low-cost, simple device, mobility and flexibility features make the sleep apps an excellent choice to support sleep. However, current sleep apps have some limitations, such as accuracy, privacy and security issues, short battery life and information quality. Frequently reported design guidelines for developing sleep apps are user-centered, evidence-based, theory-based, engagement, feedback, accessible format and social connectedness. We did not find much information about the user requirements of sleep apps for different populations. Future research is needed to determine the user requirements for diverse populations.

## Author contributions

AA and OM conceived and designed the review. JW collected data and conducted the initial analysis with the help of AA. JW and AA wrote the manuscript, which was reviewed, and revised by OM. All authors contributed to the article and approved the submitted version.

## Conflict of interest

The authors declare that the research was conducted in the absence of any commercial or financial relationships that could be construed as a potential conflict of interest.

## Publisher's note

All claims expressed in this article are solely those of the authors and do not necessarily represent those of their affiliated organizations, or those of the publisher, the editors and the reviewers. Any product that may be evaluated in this article, or claim that may be made by its manufacturer, is not guaranteed or endorsed by the publisher.
